# Acute Response *in vivo* of a Fiber-Optic Sensor for Continuous Glucose Monitoring from Canine Studies on Point Accuracy

**DOI:** 10.3390/s100807789

**Published:** 2010-08-20

**Authors:** Kuo-Chih Liao, Shih-Chieh Chang, Cheng-Yang Chiu, Yu-Hsiang Chou

**Affiliations:** 1 Graduate Institute of Biomedical Engineering, National Chung-Hsing University, 250 Kuo-Kuang Rd., Taichung City, 40227, Taiwan; E-Mail: micchou@hotmail.com (Y.-H.C.); 2 Department of Veterinary Medicine / Veterinary Medical Teaching Hospital, National Chung-Hsing University, 250 Kuo-Kuang Rd., Taichung City, 40227, Taiwan; E-Mails: scchang@dragon.nchu.edu.tw (S.-C.C.); d9838005@mail.nchu.edu.tw (C.-Y.C.)

**Keywords:** *in vivo* glucose monitoring, fiber-optic sensor, point accuracy, Clarke error grid analysis, calibration

## Abstract

The objective of this study was to evaluate the acute response of Sencil^™^, a fiber-optic sensor, in point accuracy for glucose monitoring *in vivo* on healthy dogs under anesthesia. A total of four dogs with clinically normal glycemia were implanted with one sensor each in the chest region to measure the interstitial glucose concentration during the ovariohysterectomy procedure. The data was acquired every 10 seconds after initiation, and was compared to the concentration of venous plasma glucose sampled during the surgery procedures for accuracy of agreement analysis. In the four trials with a range of 71–297 mg/dL plasma glucose, the collected 21 pairs of ISF readings from the Sencil™ and the plasma reference showed superior dispersion of residue values than the conventional system, and a linear correlation (the Pearson correlation coefficient is 0.9288 and the y-intercept is 14.22 mg/dL). The MAD (17.6 mg/dL) and RMAD (16.16%) of Sencil™ measurements were in the comparable range of the conventional system. The Clarke error grid analysis indicated that 100% of the paired points were in the clinically acceptable zone A (61.9%) and B (38.1%).

## 1. Introduction

The ultimate goal of glucose sensing development is to fulfill the requirements of a complete artificial pancreas, with a continuous *in vivo* monitoring for fine-tuning an insulin pump by closed-loop control [1,2]. Such a treatment could dramatically reduce mortality and complications from the chronic metabolic fluctuations in either hyperglycemia or hypoglycemia, and alleviate the burden of care delivery cost [3,4]. Above all, this hypothetical system could significantly improve the living quality of millions of diabetes sufferers worldwide to resemble the life of healthy individuals. In the current clinical environment, insulin delivery technology (insulin formulation and pump designs) is well developed, however glucose sensors for continuous *in vivo* monitoring remain problematic, especially with regards to the kinetic response in the hypoglycemia region [5,6]. Presently, intermittent self-monitoring of blood glucose (SMBG) is the clinical method of choice, which provides point accuracy and has been verified by Clarke error grid analysis (conventional devices have 95–99% accuracy) [7]. However, an ideal glucose sensor for an artificial pancreas should provide clinical accuracy not only in the aspect of absolute glucose value at a specific time (point accuracy) but also the rate and direction of concentration change (rate accuracy) [7]. It has been noted that physiological insulin secretion from β-cells (insulin secretion portion in the pancreas) has a biphasic response toward a step change of glucose, which can be simulated by a close-loop feedback controlled insulin pump with a PID controller [6,8,9]. The PID controller will be adjusted by three coefficients: proportional to the error (measured glucose concentration minus target concentration); the area under the curve (integral) of the error; rate of change (derivative) of the error. The point accuracy of the signal-feedback sensor has the dominant influence on the “P” component (control gain); on the other hand, the “I” (direction of change) and “D” (rate of change) components will be counted on a sensor with reliable rate accuracy.

**Figure 1.**
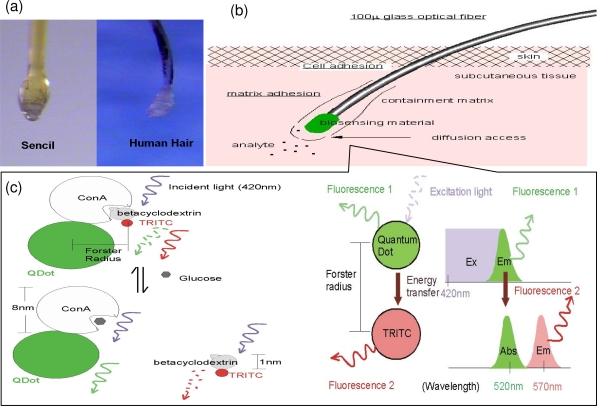
Illustration of the Sencil™ system for glucose sensing. (a) Similarity of shape and size between Sencil™ and human hair with attached follicle (white bar = 100 μm). (b) Sensor components and relationships to tissue *in vivo*. (c) Changes in FRET between fluorophores covalently immobilized on the flexible PEG matrix depend on changes in the distance between them, which in turn depends on the competitive natural affinity between Con A and various saccharides such as betacyclodextrin and glucose. The figure is modified with permission from [10], published by Biosensors Bioelectronics, 2008.

Sencils™ are a family of fiber-optic sensors, capable of providing analyte monitoring *in vivo* for several weeks [10,11]. The key element is a chronically implantable and human hair-like optical fiber (Figure 1a) that permits reliable spectroscopic measurement of chemical reactions from the subcutaneous end of the fiber (Figure 1b). The technology can benefit not only clinical applications, but also veterinary medicine and basic molecular biology research. Preliminary animal implantation studies have demonstrated good chronic biocompatibility and durability [11]. Its first application was to measure glucose based on changes in fluorescence resonance energy transfer (FRET) between fluorophores bound to betacyclodextrin and concanavalin A (Con A) in a polyethylene glycol (PEG) matrix attached to the implanted end of the fiber (Figure 1c). *In vitro* experiments demonstrated a rapid and linear relationship between the ratio of the two fluorescent emissions and concentration of glucose in saline for the physiological range of concentrations (0–500 mg/dL) over seven weeks [10]. The preliminary results confirm that applying ratio as the concentration index makes the reversible assay insensitive to variability of the photonic coupling or deterioration of the sensing materials over time. It could allow the device possess an internal standard without the necessity to calibrate daily based on the SMBG reading.

Like most of the minimal and noninvasive designs for continuously *in vivo* glucose monitoring [12–15], Sencil™ measures glucose in the interstitial fluid (ISF) compartment of skin to estimate plasma glucose. Although the concentration of the plasma glucose is the clinical index in identifying and managing diabetes, the ISF glucose concentration has a closer correlation to the development of complications on the peripheral tissues through the regional cell-level activities [16]. The ISF glucose and plasma glucose are expected to have different steady-state concentrations and kinetic responses under conditions with all the physiological barriers [17,18], but the detection is the main objective due to its ease of use and safe accessibility. The estimation of plasma glucose from ISF glucose introduces discrepancy from the sensor detection and time delay of the concentration gradient between the blood pool and ISF through the capillary vessel wall. The concentration gradient between ISF and plasma can be approximated by the two-compartment model [17,19,20]. The main challenge of the detection accuracy is on the design to minimize the sensor delay and the establishment of a valid and efficient *in vivo* calibration [12,13,16,20,21].

The aim of this paper was to study the acute response of Sencil™ for glucose detection in a clinical environment and address the follow questions: (1) how long does the sensor settled in tissue after implantation for short-term applications, and (2) how accurate is the measurement from acute response under *in vitro* calibration, and (3) how accurate is the acute response to estimate plasma glucose from the ISF detection? The information is crucial to establish foundation for further *in vivo* testing in elaborating the sensor accuracy of full working range through glucose clamp experiments, and chronic *in vivo* performance.

## 2. Experimental Section

### 2.1. Canine Subjects

Four clinical healthy dogs scheduled for ovariohysterectomy were selected for the study. All dogs had a record of normal glycemia when admitted. Physical examination of each dog was performed by a board-certificated veterinary surgeon or veterinary resident to confirm the subject’s health. The hematological profile (WBC, RBC, Hb., Hct., MCV, MCH, MCHC, Thromob., Parasite) and bloodchemistry profile (AST/STOG, ALT/SGPT, LDH, CK, Alk. P’tase, Glucose, BUN, Creatinine, T. protein, Albumin, Calcium, Phosphorous) were verified within normal limits by Hitachi 7050 prior the induction of anesthesia. The study protocol was approved by the National Chung-Hsing University Institutional Animal Care and Use Committee (IACUC Approval No.: 98-19), and consent was obtained from owners of all participating subjects.

### 2.2. Anesthesia

Anesthesia was induced with intravenous administration of medetomidin 125 mcg/m^2^ and propofol 2 mg/kg. Dogs were endotracheally intubated and anesthesia was maintained with inhalation of isoflurane in oxygen (1% to 2.5% isoflurane). The isoflurane vaporizer was adjusted as necessary to maintain an appropriate anesthetic status. All dogs received an IV infusion of lactated Ringer’s solution (Dog #1–3) or dextrose-lactated Ringer’s solution (Dog #4) in the flow rate of 10 mL/kg/h, throughout the anesthesia periods.

### 2.3. Sensor Placement and Spacial Arrangements

**Figure 2.**
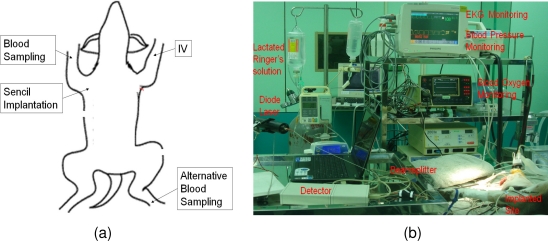
Illustration of the relative spacial positions for implantation, blood sampling and IV infusion on each subject (a). Arrangement of devices around the operation table (b).

The relative spacial positions of implantation site, blood sampling sites, IV injection site are shown in Figure 2. A Vasofix catheter was placed on the right foreleg for following blood samplings. The IV infusion was flowed through the catheter on the left foreleg. The Sencil™ was implanted on the chest about 10 cm away from the blood sampling site. The arrangement can avoid the accidental removal of the sensor probe from the skin, due to the forced movement of limb during sampling or a relatively more dramatic involuntary movement from leg (or another body part) under anesthesia.

After induction of anesthesia, the dogs were managed to supine position on the surgery table with restraints on the end of their four limbs. Hair in the chest and abdomen regions was shaved and those areas were cleaned with scrubbing chlorhexidine and alcohol. The detection end of the optical fiber (with hydrogel attached) was introduced percutaneously by a disposable sterilized gauge 23 needle in a shallow angle along the surface of the skin. The needle was pulled out off skin when the sensor probe was placed about 1–2 cm deep. The external end of the optical fiber was connected to the portable spectrophotometer system [10] through a SMA type connector. Dermabound was applied on the implantation site (for all subjects), and portion of the optical fiber extended out of skin was adhered on the skin surface with elastic adhesive tape (Dog #1–3 only, not on Dog #4) to secure the implantation. All the shank of the optical fiber, which was protruding out of the skin, was covered with aluminum foil to shield the detection from the ambient light.

### 2.4. *In Vitro* Sensor Calibration

**Figure 3.**
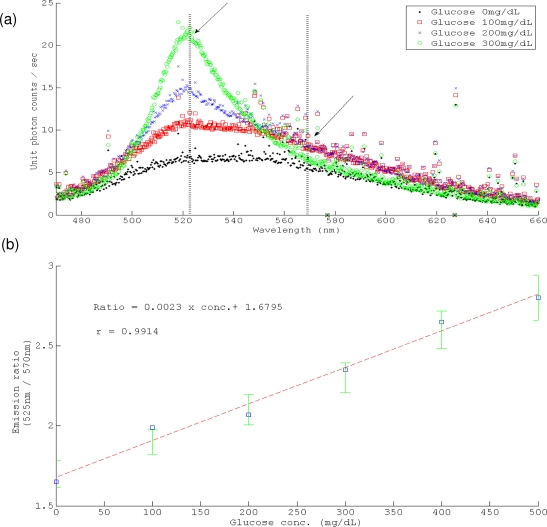
Sensor calibration from the linear fitting result of *in vitro* measurements. (a) Absolute emissions at 525 nm and 570 nm (arrows) were applied to calculate the ratio for indicating glucose concentration. (b) Linear correlation between the emission ratios and the corresponding glucose concentrations. The standard deviations (from the six sensors manufactured in the same batch) of the calibration values at corresponding glucose concentrations are shown as green error bars.

Six sensors were made in the same batch, and four of them were applied in the study during a time-span of five months. Each of them was stored in a dry sterilized pouch under the conditions specified in [10] before the study. The calibration curve was obtained from the sensor in 0, 100, 200, 300, 400, 500 mg/dL glucose salines (pH 7.4 phosphate buffer) one day prior to the implantation. The detected glucose concentration was calculated from the ratio of the two absolute value of fluorescence at their maximal emission wavelength (indicated by the arrows in Figure 3a). The conversion was based on least square linear fitting of the results. The characterization of the sensor for Dog #1 study is shown in Figure 3b, and the other sensors had similar fitting slopes and y-intercepts with the differences were less than 5%. The standard deviation of the calibration value in each corresponding glucose concentration is shown in Figure 3b.

### 2.5. Initiation and Processing of Monitoring

During *in vivo* monitoring, the absolute emissions (unit photon counts) at 525 nm and 570 nm (the maximal emission wavelength of the two fluorophores) were acquired every 10 seconds as two analog signals. Those analog signals were digitized and processed on a laptop computer installed with OOIBase 32 software through a USB connection to calculate the emission ratio (525 nm/570 nm) at each acquisition (data point). The data point which was recorded at the time of venous blood sampling was applied to be compared with the plasma reading.

After implantation, both maximal emissions at corresponding wavelengths were observed to have a significant 2–3 fold decline in the four separate studies. This could be explained by two optical events: firstly, the ambient light contributed partially in the detected signal before implantation. It had a board emission spectrum from 450 nm to 800 nm, and its intensity at 525 nm was about three times the intensity at 570 nm. Secondly, more excitation was backscattered at the interface of hydrogel with the surrounding air before implantation. The reflection of normal incidence on the interface can be calculated from equation 1 to demonstrate a 10-fold increase in air (n = 1.0003) than in a water rich environment (n = 1.33, such as in cell clusters) when the refractive index of hydrogel is assumed to be 1.45. The dramatic change in emission intensity can be applied as index to check if the device falls out of skin during experiment.

(1) $$R={\left(\frac{n-n_{\mathrm{hydrogel}}}{n+n_{\mathrm{hydrogel}}}\right)}^{2}$$

**Figure 4.**
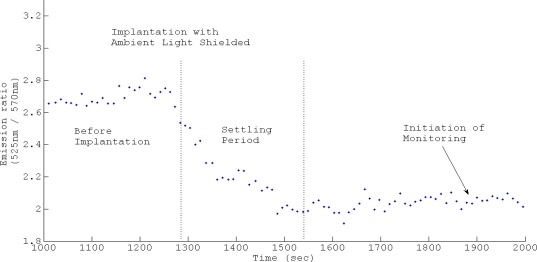
Fluorescence emission ratio change trend before and after implantation.

Figure 4 demonstrates the relatively mild transition (∼30%) of emission ratio before and after implantation from study one while the other three studies shared the similar trends. The whole process of settling periods in the four studies were consistently in the range of 3–6 minutes, which is similar to the temporal response of Sencil™ to a step change of glucose from the *in vitro* studies [10] and provides the evidence to support the hypothesis that diffusion of bio-fluid into the matrix could gradually reduce the refractive index of hydrogel, and account for the change. For further *in vivo* studies, all monitoring should be initiated at approximately 10 minutes after the implantation to avoid the interference from the transition process based on the present observations.

## 3. Results and Discussion

### 3.1. Canine Subjects

Four dogs, three mixed-breed and one Golden Retriever were included in this research. The mean body weight was 20 kg (from 13 to 32 kg). All subjects underwent standard ovariohysterectomy in the abdomen region and anesthesia for 125 to 186 minutes. The venous blood samples were drawn (1 mL) from the foreleg (specified in Figure 2) in the interval of 13 to 56 minutes without interfering with the progress of the surgery. A total of 21 pairs of ISF readings from Sencil™ and plasma glucose reference from the Hitachi 7050 instument were collected for analysis.

### 3.2. Canine Studys #1–3

**Figure 5.**
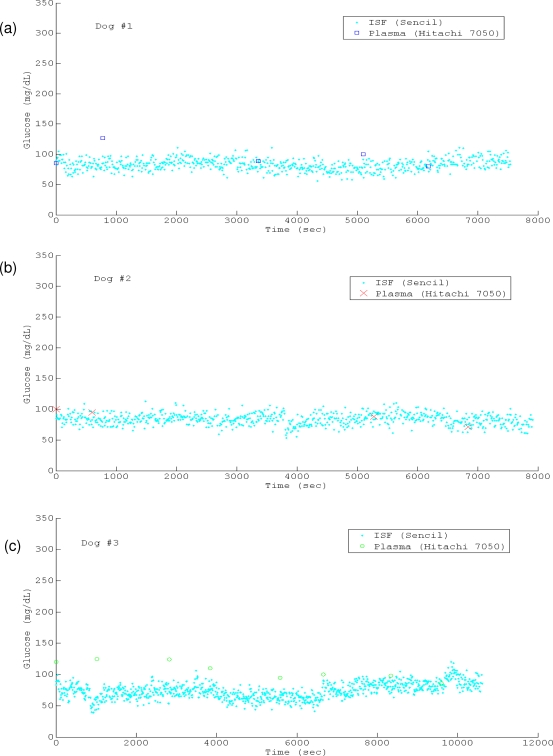

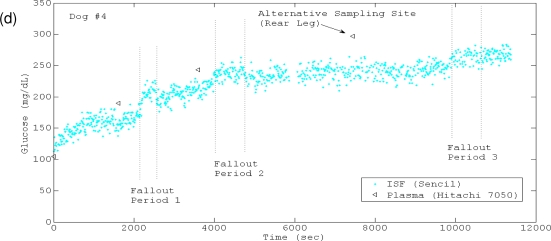
Profiles of the ISF reading from Sencil™ and plasma reference from Hitachi 7050. (a) case study #1 (b) case study #2 (c) case study #3 (d) case study #4.

The studies of Dogs #1–3 had similar experimental conditions, including the glucose-free infusions, which started 5–15 minutes prior the initiation of ISF glucose monitoring by Sencil™. The detected ISF glucose of Dogs #1–2 fluctuated mostly in the normal canine glycemia range (plasma glucose 65–110 mg/dL) with less than 10% of detections below or above the range (Figures 5a and 5b). The corresponding mean (standard deviation) values of the two trials were 82.9065 (±9.5001) and 83.6616 (±9.2934) mg/dL. However, the ISF glucose level of dog #3 appeared to be maintained at the lower limit of the normal glycemia during the first 2/3 of the anesthesia period (Figure 5c). Its mean (standard deviation) value was 74.0334 (±12.5593), and it showed the largest discrepancy (the mean absolute difference, MAD, was 19.75 mg/dL calculated by Equation 2, or the relative mean absolute difference, RMAD, calculated by Equation 3) between the ISF reading and plasma reference in the three trials (the corresponding MAD and RMAD values of trial 1 and 2 were 10.39 mg/dL, 9.75% and 7.01 mg/dL, 6.64%).

(2) MAD=mean(|Plasma−ISF|)

(3) $$\mathrm{RMAD}=\mathrm{mean}(\frac{\left|\mathrm{Plasma}-\mathrm{ISF}\right|}{\mathrm{Plasma}})\times 100\%$$

### 3.3. Canine Study #4

Case study #4 chose a glucose-included infusion to induce hyperglycemia during monitoring (Figure 4d). The ISF glucose level climbed up all the way from normal glycemia range and eventually reached a plateau around 300 mg/dL detected by Sencil™. The anchorage of Sencil™ was enhanced by Dermabond only in this study, without the application of elastic adhesive tape as the other three trials. The probe fell out three times during the monitoring procedure due to involuntary movements of the subject. A 2–3 fold emission intensity was observed during those periods, however the corresponding emission ratio was only slightly elevated (<10%). The Vasofix catheter for blood sampling formed a blood clot that could not be fixed with heparin injection, so the last venous blood sample was collected from the rear leg of the subject. A plasma reading at 637 mg/dL was collected 26 minutes before the last sampling with the venous blood drawn from the IV-infused limb, and was not included in the following analysis. The MAD and RMAD values of this case were 41.25 mg/dL, 18.49%.

### 3.4. Accuracy of Agreement between Sencil Detections and Plasma Reference for Clinical Application

**Figure 6.**
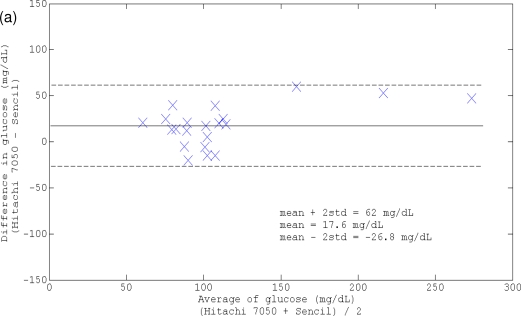

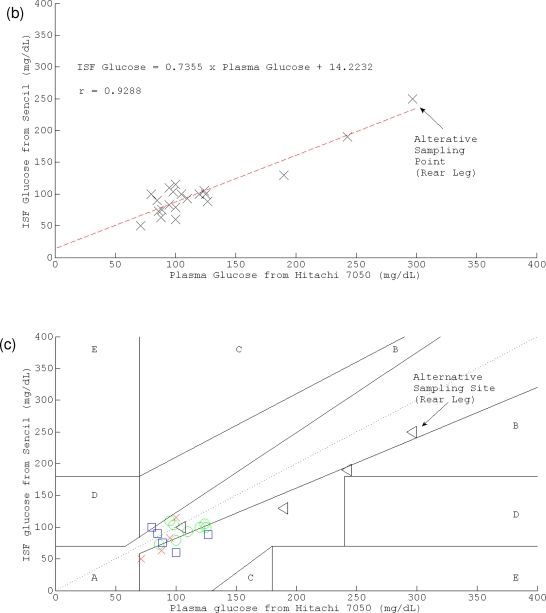
Accuracy of agreement between Sencil reading and plasma reference: (a) Bland-Altman method analysis; (b) least square linear fitting result; (c) Clarke error grid analysis.

The accuracy of agreement between Sencil™ measurements and plasma reference for *in vivo* glucose monitoring was assessed by the method of residues, linear correlation, mean absolute difference, and Clarke error grid analysis. In the four trials with a range of 71–297 mg/dL plasma glucose, the collected 21 pairs of ISF reading from Sencil™ and plasma glucose reference from Hitachi 7050 showed that Sencil™ slightly overestimated the plasma reference value, which was indicated by the positive mean of difference (mean = 17.6 mg/dL). The MAD (17.6 mg/dL) and RMAD (16.16%) of Sencil™ measurements were in the similar range of the conventional Medtronic continuous *in vivo* monitoring system [26]. However, the dispersion of values (expressed as mean ± 2 std) around the mean difference was lower for Sencil™ (45 mg/dL, shown in Figure 6a) than with the conventional system (84 mg/dL). A linear correlation (the Pearson correlation coefficient is 0.9288 and the y-intercept is 14.22 mg/dL) between ISF reading and plasma reference is shown in Figure 6b. The Clarke error grid analysis indicated that 100% of the points are in the clinical acceptable zone A (61.9%) and B (38.1%) as shown in Figure 6c. The results of accuracy agreement analysis implied the potential of the sensor for clinical application from limited number of trials. However, a relative larger discrepancy was observed when the ISF glucose had more significant shift such as case studies #3 and 4, which could be resolved in the following directions:

First of all, the study did not correct for the diffusion lag which results from the concentration gradient between the blood pool and the ISF compartment. Typically, ISF glucose falls ahead of plasma glucose when glycemia declines due to the regional uptake (triggered by endogenous insulin), and lags behind plasma glucose when glycemia increases. The time constant of the two phases can be derived from the two compartment model through a series of glucose clamp studies, endogenous hypoinsulinemic tests and endogenous hyperinsulinemic tests [17,19,20]. Secondly, the ISF glucose is calculated from the absolute emission intensity at 525 nm and 570 nm (Figure 3a), the theoretical emission maximal wavelengths of the two fluorophores (Qdot and FITC) in the sensing matrix. However, the peak amplitudes of each are influenced by the skirts due to overlap of the two emissions spectra. The problem is exacerbated for high glucose concentrations (>300 mg/dL) in which Qdot fluorescence is less quenched by FRET and the emission peak of TRITC is completely obscured by the Qdot emission. Hypothetically, estimating the area-under-the-curve attributable to each fluorophore and filtered by numerical signal processing tools, such as independent component analysis could be the potential remedy for this problem. Thirdly, the implanted component of the sensor could induce a foreign body reaction that changes the local concentration of the analyte or that blocks diffusion of the analyte to the transduction point for chronic application [23–25]. Immediately upon contact with extracellular fluid (especially blood), proteins and other chemicals will adsorb onto and diffuse into the sensor, which may block protective or analyte-selective membrane; and a fibrous capsule will be formed progressively during post-implant days 4–12, potentially blocking diffusion of the analyte. The capsule may have low or fluctuating concentrations of oxygen, glucose and other metabolites depending on the ratio of metabolic activity of the capsular cells to the circulation provided by the interrupted and reforming capillary bed. It might result in detection drift. Although the Sencil™ implant has been verified to form a stable interface with the surrounding tissue [11], the effect of the relatively thin encapsulation should be tested in a chronic monitoring study. In this study, we observed the acute response on glucose sensing without a stable device/issue interface. The condition is similar to all of the present FDA-approved continuous monitoring systems (Guardian^®^ system and Paradigm^®^ system of Medtronic, and Seven Plus system of Dexcom start measurement at 2 hours after insertion; Freestyle Navigator^®^ of Abbott begins at 10 hour). They all need several *in vivo* calibrations against plasma glucose at specific timeframes in their lifetimes (3–7 days) to adjust the tissue responses. The preliminary result of this study suggested that the acute response of the fiber-optic sensor possesses reasonable accuracy for clinical application without *in vivo* calibration. According to the recent literature reports [24,25] on the tissue response to needle type enzymatic continuous glucose sensors (general design scheme of all the marketed systems mentioned above), the detection was not apparently affected in the early phase response to the trauma. It was the fibrous capsule formed in the end stage of foreign body reaction caused the most disturbances in glucose sensing, and eventually the function loss of the device. We have demonstrated that the small size and biocompatibility of the fiber-optic sensor formed relatively much thinner fibrous capsule than the needle type enzymatic continuous glucose sensors [11,24], and intend to verify that the Sencil™ will have longer lifetime due to the relatively mild foreign body reaction in the future studies.

## 4. Conclusions

The present acute response study demonstrates the close correlation between ISF glucose readings from Sencil™ and plasma glucose reference values over an approximately two hours of monitoring after calibration based on *in vitro* glucose saline readings. Further point accuracy analysis over the potential working range, rate accuracy analysis and chronic monitoring experiment will identify the feasibility of Sencil™ for continuous glucose monitoring *in vivo*, or ultimately as a the closed-loop feedback input for an artificial pancreas.

## References

[1] Pickup JC (1993). *In vivo* glucose monitoring: Sense and sensorobility. Diabetes Care.

[2] Wilson GS, Reach G (1992). Diabetes and glucose sensors. Anal. Chem.

[3] Diabetes Control and Complication Trials Research Group (1993). The effect of intensive treatment of diabetes on the development and progression of long-term complications in insulin-dependent diabetes mellitus. N. Engl. J. Med.

[4] UK Prospective Diabetes Study Group (UKPDS 33) (1998). Intensive blood-glucose control with sulphonylureas or insulin compared with conventional treatment and risk of complications in patients with type 2 diabetes. Lancet.

[5] Wentholt I, Hoekstra J, DeVries J (2006). A critical appraisal of the continuous error grid analysis. Diabetes Care.

[6] Steil GM, Panteleon AE, Rebrin K (2004). Close-loop insulin delivery—The path to physiological glucose control. Advan. Drug Delivery Rev.

[7] Clarke WL (2005). The original Clarke error grid analysis (EGA). Diabetes Technol. Ther.

[8] Steil GM, Rebrin K, Mastrototaro JJ (2006). Metabolic modeling and the close-loop insulin delivery problem. Diabetes Res. Clin. Pract.

[9] Steil GM, Rebrin K, Janowski R, Darwin C, Saad MF (2003). Modeling *β*-cell secretion—Implications for closed-loop glucose homeostasis. Diabetes Technol. Ther.

[10] Liao KC, Hogen-Esch T, Richmond FJ, Marcu L, Clifton W, Loeb GE (2008). Percutaneous fiber-optic sensor for chronic glucose monitoring *in vivo*. Biosensor. Bioelectron.

[11] Liao KC, Hogen-Esch T, Richmond FJ, Marcu L, Loeb GE Design and Fabrication of Disposable, Percutaneous Chemical Sensors.

[12] Garg S, Zisser H, Schwartz S, Bailey T, Kaplan R, Ellis S, Jovanovic L (2006). Improvement in glycemic excursions with a transcutaneous, real-time continuous glucose sensor: A randomized controlled trial. Diabetes Care.

[13] Garg SK, Potts RO, Ackerman NR, Fermi SJ, Tamada JA, Chase HP (1999). Correlation of fingerstick blood glucose measurements with GlucoWatch biographer glucose result in young subjects with type 1 diabetes. Diabetes Care.

[14] Shichri M, Sakakida M, Nishida K, Shioda S (1998). Enhanced, simplified glucose sensors: Long-term clinical application of wearable artificial endocrine pancreas. Artific. Organ.

[15] Bindra DS, Zhang Y, Wilson GS, Sternberg R, Thevenot DR, Moatti D, Reach G (1991). Design and *in vitro* studies of a needle-type glucose sensor for subcutaneous monitoring. Anal. Chem.

[16] Boyne MS, Silver DM, Kaplan J, Saudek CD (2003). Timing of changes in interstitial and venous blood glucose measured with a continuous subcutaneous glucose sensor. Diabetes.

[17] Aussedat B, Dupire-Angel M, Gifford R, Klein JC, Wilson GS, Reach G (2000). Interstitial glucose concentration and glycemia: Implications for continuous subcutaneous glucose monitoring. Amer. J. Physiol-Endocr. Metab.

[18] Kulcu E, Potts RO, Tamada JA, Lesho MJ, Reach G (2003). Physiological differences between interstitial glucose and blood glucose measured in human subjects. Diabetes Care.

[19] Rebrin K, Steil GM (2000). Can interstitial glucose assessment replace blood glucose measurement?. Diabetes Technol. Ther.

[20] Rebrin K, Steil GM, van Antwerp WP, Mastrototaro JJ (1999). Subcutaneous glucose predicts plasma glucose independent of insulin: Implications for continuous monitoring. Am. J. Physiol-Endocrinol. Metab.

[21] Panteleon AE, Rebrin K, Steil GM (2003). The role of the independent variable to glucose sensor calibration. Diabetes Technol. Ther.

[22] Hecht E (2002). The propagation of light. Optics.

[23] Labow RS, Erfle DJ, Santerre JP (1995). Neutrophil-mediated degradation of segmented polyurethanes. Biomaterials.

[24] Heninger N, Woderer S, Kloetzer HM, Staib A, Gillen R, Li L, Yu X, Norbert G, Kraenzlin B, Pill J (2007). Tissue response to subcutaneous implantation of glucose-oxidase-based glucose sensor in rats. Biosensor. Bioelectron.

[25] Klueh U, Kaur M, Qiao Y, Kreutzer DL (2010). Critical role of tissue mast cells in controlling long-term glucose sensor function *in vivo*. Biomaterials.

[26] Guerci B, Benichu M, Floriot M, Jellimann S, Bohme P, Drouin P, Durain D (2003). Clinical performance of CGMS in type 1 diabetic patients treated by continuous subcutaneous insulin infusion using insulin analog. Diabetes Care.

